# What you don’t know can still hurt you - underreporting in EU pesticide regulation

**DOI:** 10.1186/s12940-022-00891-7

**Published:** 2022-09-05

**Authors:** Axel Mie, Christina Rudén

**Affiliations:** 1 Department of Environmental Science, Stockholm University, 10691 Stockholm, Sweden; 2 Department of Clinical Science and Education, Karolinska Institutet, Södersjukhuset, 11883 Stockholm, Sweden; 3 Swedish University of Agricultural Sciences (SLU), Centre for Organic Food and Farming (EPOK), Ultuna, Uppsala, Sweden

**Keywords:** Glyphosate, Glyphosate trimesium, Developmental neurotoxicity, Pesticide dossier, Underreporting, Reporting bias

## Abstract

The safety evaluation of pesticides in the European Union (EU) relies to a large extent on toxicity studies commissioned and funded by the industry. The herbicide glyphosate and four of its salts are currently under evaluation for renewed market approval in the EU. The safety documentation submitted by the applicant companies does not include any animal study regarding developmental neurotoxicity (DNT) that is compliant with test guidelines. For a fifth salt, not included in the present application for re-approval, such a DNT study was sponsored by one of the applicant companies in 2001. That study shows an effect of that form of glyphosate on a neurobehavioural function, motor activity, in rat offspring at a dose previously not known to cause adverse effects. Counter to regulatory requirements, these effects were apparently not communicated to authorities in EU countries where that form of glyphosate was authorised at that time. That DNT study may also be relevant for the ongoing assessment of glyphosate but was not included in the present or previous applications for re-approval.

In this commentary, we highlight that it is the responsibility of the industry to evaluate and ensure the safety of their products, taking all available scientific knowledge into account. We argue that the legal obligation for industry to submit all potentially relevant data to EU authorities is clear and far-reaching, but that these obligations were not fulfilled in this case. We claim that authorities cannot reliably pursue a high level of protection of human health, if potentially relevant evidence is withheld from them. We suggest that a retrospective cross-check of lists of studies performed by test laboratories against studies submitted to regulatory authorities should be performed, in order to investigate the completeness of data submitted to authorities. We further suggest that future toxicity studies should be commissioned by authorities rather than by companies, to improve the authorities’ oversight over existing data and to prevent that economic conflicts of interest affect the reporting of study results and conclusions.

## Background

### Chemical plant protection products require premarket testing and assessment

Chemical plant protection products are used on crops to e.g. kill weed and prevent disease and infestation with different pests. The substance(s) that are added to the product to enable this effect are called active substances. Since many plant protection products are designed to be toxic to target organisms, and used in high volumes in food production, their marketing and use are tightly regulated. Starting in the 1990’s, the safety evaluation of active substances was harmonised between EU member states, and a common procedure gradually replaced previous independent assessments by each country.

Before a plant protection product can be put on the market, it has to be tested for efficacy, i.e. its pest controlling abilities, for potential “side effects”, i.e. adverse effects to non-target species, including humans, and for its environmental fate and behaviour [1, 2]. The manufacturing industry is responsible for generating, evaluating, and submitting the information required. They usually commission studies to externally contracted laboratories.

The company seeking market approval for the active substance collects the data and their own assessment in a “dossier” and submits it to the EU member state acting as Rapporteur Member State (RMS). The RMS produces an “assessment report”. The European Food Safety Authority (EFSA) is responsible for reviewing the assessment report and produces a “conclusion”. Based on this conclusion, the European Commission decides on the approval at the EU level in agreement with member states [2]. Active substances generally get market approval for a period of 10 years. After that, renewal is possible, typically for 15 years at a time.

The EU Member States are responsible for evaluating and authorising the plant protection products containing the active substance at the national level.

One of the *approval criteria for active substances* in the EU is that “it may be expected, in the light of current scientific and technical knowledge", that its residues after proper use shall not have any harmful effects on human health, animal health, and on groundwater, and no unacceptable effects on the environment [2]. More explicit *data requirements* have been specified [3]. The first requirement is that the information in the dossier shall “be sufficient to evaluate the foreseeable risks, whether immediate or delayed, which the active substance may entail for humans, including vulnerable groups, animals and the environment and contain at least the information and results of studies” as listed in that Regulation. The second requirement is that “[a]ny information on potentially harmful effects of the active substance, its metabolites and impurities on human and animal health or on groundwater shall be included.”

These and other overarching principles are complemented by more detailed specifications regarding the evaluation of various types of effects. For example, data requirements regarding reproductive toxicity include the obligation to report any effect interfering with normal development of the offspring before and after birth. One specific type of study on developmental effects, the developmental neurotoxicity (DNT) study, evaluates effects of chemicals on the development of behavioural functions and brain morphology in offspring. DNT studies are not routinely required. The rules specify however that potential neurotoxic effects shall be carefully addressed and reported. It is also clearly stated that such investigations of developmental and reproductive toxicity “shall take account of all available and relevant data, including […] knowledge concerning structural analogues to the active substance” [3].

### Regulatory background – glyphosate (and its salts)

Glyphosate (CAS 1071-83-6) is a broad range herbicide, originally marketed in the 1970s under the trade name “Roundup”. By now many glyphosate-based products are available and it is one of the most used pesticides globally. The safety of glyphosate has been discussed as different organizations have come to different conclusions regarding the carcinogenic potential of glyphosate and its products [4].

For the first EU-wide evaluation of glyphosate, several groups of companies submitted in 1994-1996 dossiers to support its inclusion in Annex I of Directive 91/414 concerning the placing of plant protection products on the market [1]. Listing of active ingredients on Annex I was, at that time, a prerequisite for authorisation of products containing them. The glyphosate dossiers covered also its isopropylammonium, sodium, and ammonium salts. Further, one company submitted a dossier to support the inclusion of the trimethylsulfonium salt (also called ”glyphosate trimesium”). After evaluation [5], glyphosate and all of these salts were included in Annex I, without differentiation, as a single active substance with an approval until 2012 [6]. The market approval was subsequently extended until 2017 [7]. A group of companies (the “Glyphosate Task Force”, GTF) submitted a dossier to support a renewed EU approval of glyphosate in 2012. This dossier supported glyphosate as well as its isopropylammonium, potassium, and ammonium salts. Glyphosate was re-approved in 2017, and the current approval expires in December 2022.

Glyphosate trimesium was not a part of the dossier submitted in 2012, and the company that had previously pursued the trimesium salt joined the GTF. Products containing glyphosate trimesium appear to have been withdrawn from the EU market during this time. Comprehensive information is however difficult to collect since it is scattered across the member states. In Germany, the last authorisation of products containing glyphosate trimesium ended in 2004 and in Sweden it ended in 2007.

### Regulatory situation for glyphosate in the EU at present

An application for another renewal, submitted by a group of companies under the name “Glyphosate Renewal Group” (GRG), is currently being evaluated. It covers glyphosate and its potassium, isopropylammonium, ammonium, and dimethylammonium salts. The company previously pursuing glyphosate trimesium is now also a part of the GRG.

The renewal dossier was submitted in June 2020. The dossier has been evaluated by the assessment group for glyphosate (AGG) that includes experts from authorities in France, Hungary, the Netherlands and Sweden.

## DNT study not included in the EU dossier

The present dossier for glyphosate is comprehensive. However, it does not include a DNT study that is compliant with test guidelines.

A DNT study is however available for the active ingredient glyphosate trimesium [8]. It was performed in 2001, and sponsored by the company that had submitted the dossier for the trimesium salt for the first EU-wide evaluation. We learned about its existence in March 2022, and immediately informed EFSA. EFSA confirmed that the DNT study was neither included in the present nor in previous dossiers.

The DNT study has been evaluated by the U.S. EPA in 2005, and the US authority concluded that it demonstrates behavioural effects in rat offspring following exposure to maternal animals [9]. The doses were 0, 10, 25 and 100 mg glyphosate trimesium/kg body weight (bw)/day, administered to maternal animals from gestational day 7 through postnatal day (PND) 11 by gavage. The maternal lowest observed adverse effect level (LOAEL) was > 100 mg, i.e. no maternal toxicity, deemed as adverse, was noted. In the offspring, overall motor activity was decreased (45-72%) in males and females in the 25 and 100 mg groups on PND 14. These results were already recognised in the original study report from 2001 but dismissed by the test laboratory as incidental. In contrast, the U.S. EPA acknowledged these effects and set a LOAEL in offspring at 25 mg/kg bw/day and the no observed adverse effect level (NOAEL) at 10 mg/kg bw/day. So, we note that the interpretation of the data by the test laboratory differs from the U.S. authority’s interpretation; effects recognised but dismissed by the laboratory were used by the U.S. EPA to set a LOAEL. Overall, the U.S. EPA also deemed the study acceptable for regulatory use [9].

At that time, the acceptable daily intake in the EU was based on a NOAEL of 31 and 21 mg/kg bw/day for glyphosate and glyphosate trimesium, respectively; both were based on chronic toxicity studies in rats.

As citizens and scientists, we would expect that if one glyphosate salt is found to cause DNT at a dose level thought to be safe for other glyphosate salts, then it would have to be clarified, without unnecessary delay, if other variants of glyphosate share that property. This is because it could be the glyphosate molecule itself causing the effect, and use as well as human exposure to glyphosate is widespread [10]. Indeed, several provisions in EU’s pesticide regulation would serve this purpose. We see at least three violations of these provisions:

### 1. The company should have submitted the DNT study directly in 2001

Pesticide legislation in force in the EU in 2001 [1] stipulated that the holder of an authorization must “immediately notify the competent authority of all new information on the potentially dangerous effects of any plant protection product […]". Compared to other effects of glyphosate trimesium relied on in the original evaluation for setting the acceptable daily intake (ADI) [5], the effect in the DNT study was observed at a lower dose level. Therefore, a consideration of the DNT effect could have reduced the ADI already at that time.

Importantly, the DNT effect was recognised by the test laboratory in the original study report, as cited in the evaluation by U.S. EPA [9]. The observed effects were interpreted as incidental by the test laboratory and thus dismissed; nonetheless, we claim that the results as such still indicate *potentially* dangerous effects. Companies may, and often do, argue that certain observed effects are not relevant or reliable. Any final decision on dismissing apparent effects as incidental must however be made by authorities.

Considering that the effect dismissed by the test laboratory in this case was dose-dependent, consistent between sexes, and substantial in magnitude, a bias in the interpretation by the test laboratory cannot be ruled out. Needless to say, a bias in data interpretation that results in an underreporting of adverse effects will negatively affect the authorities’ ability to protect public health [11]. This is however not the main subject of the present paper.

As far as we could establish, authorities of the EU or its member states were never informed of the existence of the effects observed in the industry-sponsored DNT study of glyphosate trimesium [8], although products containing this substance were authorised at that time.

It seems therefore as if the requirement to notify the competent authorities on the observed DNT effects in 2001 has not been fulfilled.

### 2. The company should have submitted the DNT study for the ongoing re-evaluation

Glyphosate trimesium is highly water-soluble and dissociates fully in water [5] and thus also in the body. Conceptually, the observed DNT effects could then have been caused by the glyphosate molecule, or by the trimesium ion, or possibly by both in combination.

In the EU, an active substance shall be approved “if it may be expected, in the light of current scientific and technical knowledge”, that its proper use does not cause harmful effects on human health. It is the responsibility of the applicant to demonstrate this in the dossier. For the present case, at least one of the applicant companies had scientific knowledge that the glyphosate molecule, i.e. the active substance in the present dossier, was among very few potential causes of DNT effects in the study of glyphosate trimesium. It is therefore counter to the intentions of the law and the responsibilities of the applicants to assume that the glyphosate molecule has *not* caused the observed DNT effects. To make that assumption it must be established that the trimesium ion, or trimesium and glyphosate in combination, were the causes, or the glyphosate molecule has to be cleared by other evidence.

It is the responsibility of the applicant companies to appropriately make use of this scientific knowledge [2]. It can be no-one else’s responsibility because no-one else involved in the regulatory process had access to this knowledge.

In principle, the applicants could consider the glyphosate trimesium DNT study in three ways for the present assessment of glyphosate:

First, the study could be used as such to assess and characterise DNT of glyphosate and the salts currently under assessment, in conjunction with academic animal and epidemiological studies of DNT-related effects from glyphosate or its formulations [12].

Second, it could trigger the conduct of a new DNT study for glyphosate or one of the salts currently under evaluation (see also next section).

Third, it could be disregarded because the DNT observations were considered irrelevant, e.g. since they can be attributed to the trimesium ion and hence not expected to manifest with other salts. Or because the company, for some reason and in contrast to the U.S. EPA, finds the study unreliable.

It is however not clear if the applicants have considered or acted upon any of these options, as there is no reference to this study in the present glyphosate dossier.

The legislation requires companies to submit sufficient information for the evaluation of foreseeable risks, as well as “[a]ny information on potentially harmful effects of the active substance” on human health to EFSA [3]. We argue that, in whatever way the applicants decide to make use of the DNT study of glyphosate trimesium in their assessment of glyphosate, the regulatory authorities must be in a position to review that assessment and to make the final decision on how to use – or not – the existing DNT study in the present assessment of glyphosate. Therefore, the companies’ obligation to transparently report their use of the DNT study and to make the study available to EFSA is evident.

Certain differences in the toxicological profile of glyphosate and glyphosate trimesium have been recognised by EU authorities during the first evaluation over 20 years ago [5, 13]. In particular, the acute toxicity of glyphosate trimesium was substantially higher; the long-term toxicity was similar between the different forms. At that time, it was concluded that data for glyphosate trimesium should not be used for evaluation of glyphosate. This reasoning was however based on a situation where studies for each type of toxicity were available for both forms of glyphosate. The discussion did not include any reasoning or guidance on how to proceed if data indicate an adverse effect of one form of glyphosate on an endpoint where data were lacking for the other form. Pesticide regulation including the data requirements have also changed substantially since then. In any case, it would be up to EFSA, and not a company, to decide if such earlier reasoning would be applicable today in the present case.

So, it seems that this company-owned DNT study [8] should have been considered in the ongoing renewal process, and we therefore find it improper that it was omitted from the dossier submitted to EFSA.

### 3. The present dossier should have addressed DNT

For the evaluation of reproductive and developmental toxicity, there is a requirement that companies’ “[i]nvestigations shall take account of all available and relevant data, including […] knowledge concerning structural analogues to the active substance”. Also, “[p]otential neurotoxic […] effects […] shall be carefully addressed and reported” in these investigations [2].

Accordingly, in case the company sees no direct applicability of the glyphosate trimesium DNT study for other forms of glyphosate, they would still have been under obligation to consider that study and to “carefully address” DNT of those other forms of glyphosate. It is of course a matter of interpretation what it means to “carefully address” an endpoint. But one feasible option could be to commission a new DNT study. A discussion or conclusion regarding DNT of glyphosate was absent from the present dossier. Also, results from literature searches regarding effects of glyphosate on autism or ADHD risk were not included in the dossier, as already highlighted by AGG [14].

We note that the EU assessment report negated the need to perform a DNT study for glyphosate [14]. This conclusion was however not informed by results from the DNT study of glyphosate trimesium. According to the data requirements, “when indicated by observations in other studies or the mode of action of the test substance, supplementary studies or information may be required to provide information on the postnatal manifestation of effects such as developmental neurotoxicity.” [3].

## Conclusion

In our view, the legislation is clear; the DNT study of glyphosate trimesium should have been reported to authorities in the EU in 2001 and included in the current glyphosate dossier, where the applicants should have carefully addressed the potential DNT of glyphosate. None of these actions occurred. The reasoning behind these omissions, and to what extent co-applicants were informed about this matter, are unknown to us. Regardless of any strong and valid arguments that the applicants might put forward to dismiss the observed DNT effects of glyphosate trimesium, or their relevance for other forms of glyphosate, we find that they would still be required to do so explicitly in the dossier, and to inform EFSA of the data so that the regulatory authority can make its own assessment.

We would like to highlight that the analyses reported in this commentary are not intended to be understood as a detailed legal evaluation, in particular also with respect to individual companies’ responsibilities in the applicant group. Rather, our analyses are based on our understanding, as scientists and citizens, of how the EU pesticide regulatory system should work in order to live up to its high aims regarding human health protection, transparency, and of being science-based.

## Discussion

It is beyond the scope of this commentary to engage in a discussion about what regulatory consequences the DNT study will have for the market approval of glyphosate in the EU, and what consequences its non-disclosure has had for the prior approval of products containing glyphosate trimesium, as well as for prior evaluations of glyphosate. As mentioned above, we have made EFSA aware of its existence in March 2022, and the regulatory process is at present (August 2022) still ongoing.

The responsible authorities’ evaluation of the dossiers for authorization of plant protection products is comprehensive and time consuming. Nevertheless, their resources are limited. Authorities evaluate the submitted information, and they can also request additional information from the applicants, as needed. They have however no systematic way of knowing what information the applicants have access to but did not include in the dossier. The regulatory system therefore relies on trust that companies abide by the rules and submit all relevant information that is available to them. Therefore, this case has impact beyond glyphosate: It reduces our confidence that the pesticide industry submits all data on risks and hazards of their products.

Withholding relevant information is counter to taking responsibility. If the fundamental principle that companies should take full responsibility for the safety of their products is not satisfied, then this may have severe consequences for public health protection and begs to question the fundamental workings of the regulatory system.

There are some well-documented examples where industry did not disseminate scientific evidence indicating health adverse effects, e.g. tobacco [15] and per- and polyfluorinated alkyl substances (PFAS) [16]. We do not know how often this occurs, but we argue that it needs to be thoroughly investigated.

## Ways forward

We find the legislation both clear and strict in requiring a submission of all relevant data in pesticide dossiers. Compliance with these requirements can however not be efficiently confirmed. We argue that this must change.

The recent EU Transparency Rules affecting food law that were implemented in March 2021 [17] require *companies and test laboratories to notify EFSA of any study commissioned or carried out by them* to support an application for pesticide approval. As this new rule becomes fully implemented, the studies submitted to the authorities in future dossiers can be checked against the list of notified studies. Thereby, the possibility to withhold tests from the authorities will be reduced. This is an important step towards improved transparency.

In addition, *procedures should be revised so that all regulatory toxicity studies are commissioned by regulatory authorities, while still being financed by the industry*. Such an approach would reduce concerns that economic conflicts of interest are allowed to affect the interpretation and reporting of data, as indicated in the present case, and also for the insecticide chlorpyrifos [11, 18]. At the same time, authorities would gain better oversight of data availability.

However, neither of these approaches informs about undisclosed studies that already exist. We therefore propose to make use of the principles and regulations of Good Laboratory Practice (GLP). GLP sets rules for how studies are planned, performed, recorded and reported, and compliance is mandatory for industry-commissioned studies of pesticide safety that are performed for the purpose of a market approval.

Member States must perform regular inspections at testing facilities operating under GLP [19]. The information that the testing facility must provide to the inspecting authority includes *a list of the facility’s on-going and completed studies*. We propose that such lists *may be used retrospectively for cross checking against lists of studies that have been submitted to EFSA* as part of pesticide dossiers. This approach may contribute to an understanding of how often commissioned studies of apparent relevance to the safety evaluation of pesticides are omitted from dossiers submitted to EFSA. To further promote transparency and third-party scrutiny, this information should also be made publicly available.

The GLP rules apply to the safety testing of all chemicals, not just pesticides; therefore, this proposed approach for cross-checking of performed against submitted studies could also be used in other pieces of chemicals legislation within the EU.

## Data Availability

Data sharing is not applicable to this article as no datasets were generated or analysed during the current study. EU legislative documents are available at https://eur-lex.europa.eu/. Dossiers and assessment reports for the present and previous evaluation of glyphosate are available from the Open EFSA portal at https://open.efsa.europa.eu/. Certain documents from the first EU evaluation of glyphosate and glyphosate trimesium are available through the EU Commission’s “Access to documents project (Pesticides & Biocides)” at https://webgate.ec.europa.eu/dyna2/extdoc/, in particular under access request 2021/2090. (All web addresses accessed on 16th of May, 2022).

## Abbreviations

AGG: Assessment group on glyphosate
BPR: Biocidal products regulation
bw: Body weight
DNT: Developmental neurotoxicity
EFSA: European Food Safety Authority
EU: European Union
GLP: Good laboratory practice
GRG: Glyphosate renewal group
GTF: Glyphosate task force
LOAEL: Lowest observed adverse effect level
NOAEL: No observed adverse effect level
PND: Postnatal day
REACH: Registration, evaluation, authorisation and restriction of chemicals
U.S. EPA: United States Environmental Protection Agency
